# The Role of mTOR and Injury in Developing Salispheres

**DOI:** 10.3390/biomedicines11020604

**Published:** 2023-02-17

**Authors:** Rimah Saleem, Guy Carpenter

**Affiliations:** 1 College of Medicine, Alfaisal University, Al Takhassousi, Riyadh 11533, Saudi Arabia; 2 Salivary Research, Centre for Host Microbiome Interactions, Faculty of Dental, Oral and Craniofacial Sciences, King’s College London, London SE1 9RT, UK

**Keywords:** salivary glands, salispheres, mTOR, LiCl, ligation

## Abstract

Salispheres are the representative primitive cells of salivary glands grown in vitro in a nonadherent system. In this study, we used the ligation model for salisphere isolation after seven days of obstruction of the main excretory duct of the submandibular gland. The mammalian target of rapamycin (mTOR) is a critical signalling pathway involved in many cellular functions and is suggested to play a role in atrophy. We determined the role of mTOR and injury in the formation and development of salispheres. Morphological assessments and Western blot analysis illustrated how mTOR inhibition by rapamycin impaired the assembly of salispheres and how indirect stimulation of mTOR by lithium chloride (LiCl) assisted in the expansion of the salispheres. The use of rapamycin highlighted the necessity of mTOR for the development of salispheres as it affected the morphology and inhibited the phosphorylation of the eukaryotic translation initiation factor 4E-binding protein (4e-bp1). mTOR activity also appeared to be a crucial regulator for growing salispheres, even from the ligated gland. However, atrophy induced by ductal ligation resulted in a morphological alteration. The phosphorylation of 4e-bp1 and S6 ribosomal protein in cultured salispheres from ligated glands suggests that mTOR was not responsible for the morphological modification, but other unexplored factors were involved. This exploratory study indicates that active mTOR is essential for growing healthy salispheres. In addition, mTOR stimulation by LiCl could effectively play a role in the expansion of salispheres. The impact of atrophy on salispheres suggests a complex mechanism behind the morphological alteration, which requires further investigation.

## 1. Introduction

Tissue homeostasis and regeneration are vital processes regulated by the asymmetric division of resident adult stem cells [1,2]. Ligation and irradiation models, along with label-retaining studies, have proven the existence of stem/progenitor cells in the ductal compartment of salivary glands [3,4,5,6,7,8,9]. Research on salivary gland regeneration is growing considerably. The permanent loss of salivary gland function due to radiotherapy treatment in head and neck cancer patients dramatically affects quality of life. In such cases, patients become more susceptible to oral infections and difficulties in chewing, swallowing, and speech [10,11,12]. The development of the in vitro sphere culture system in 2008 by Lombaert et al. facilitated the exploration of the characteristics and the regenerative capacity of primitive cells [8]. The term ‘salisphere’ refers to the in vitro-cultured spherical clusters representing salivary gland primitive cells. Human and mouse salivary glands can develop salispheres that express several stem cell markers such as c-kit and can form ductal-like structures in the matrigel [8,13].

Biological processes, including tissue homeostasis and regeneration, require sophisticated communication and regulation by different signalling networks. At the cellular level, signalling pathways are essential for proliferation and growth. Since salispheres are derived from adult salivary gland tissues, they are usually limited in number and are reduced with age [14,15]. Several studies have explored the importance of signalling molecules such as Rho-associated protein kinase (ROCK), insulin growth factor 1 (IGF1), and canonical Wnt/β-catenin signalling for the expansion of salispheres [1,16,17]. Although the expression of stem cell markers in primitive cells indicates higher stem cell potential, it has been suggested that the administration of growth factors could improve the expression levels of the stem cell markers [16,18].

Therefore, we are interested in the mammalian target of rapamycin (mTOR), a highly conserved kinase that consists of the following two complexes: mTORC1 and mTORC2. It is widely involved in cell growth, proliferation, differentiation, and autophagy. mTOR is controlled by several effectors such as nutrients, energy, growth factors, and the canonical Wnt/β-catenin signalling pathway. The activation of mTOR is determined by the phosphorylation of the eukaryotic translation initiation factor 4E-binding protein (4e-bp1) and the phosphorylation of the ribosomal protein S6 kinase 1 (S6K1) [19,20,21,22,23].

In this exploratory study, mTOR and injury were chosen as possible factors regulating the growth, affecting the morphology, and enhancing the expansion of salispheres. Hence, this study aims to investigate the role of mTOR in the development and expansion of salispheres from unoperated and injured submandibular glands in mice.

## 2. Materials and Methods

### 2.1. Animals

In total, 25 adult female ICR mice were obtained from Charles River Laboratories (Margate, UK), weighing 25–30 g upon arrival. Mice were housed in groups of four and supplied with food and water ad libitum. A 12 h light–dark cycle was maintained at a constant temperature of 20–22 °C, and environmental enrichment (tunnels and nesting material) were provided in each cage. Animals were kept for one week under clean conditions to acclimatise to their new environment before applying any experimental procedure. All animal procedures were conducted under the UK Home Office Animal (Scientific Procedures) Act 1986. All procedures were carried out under aseptic conditions. Animal procedure was performed after the approval of the Home Office and Kings’ College London Biological Safety committee under reference number PA83EDD4A7 in October 2018.

### 2.2. Ligation of Submandibular Glands

Ligation was initially performed by anaesthetising mice with xylazine (5 mg/Kg)/ketamine (25 mg/Kg) i.p injections. After reaching a sufficient depth of anaesthesia, a 0.5 cm skin incision was made in the centre of the neck. Blunt dissection was performed, where the fat surrounding the salivary glands was dispersed, and the left submandibular excretory duct was ligated by a 6-0 Ethicon suture (Johnson and Johnson Intl, Brussels, Belgium). Following surgery, the neck was sutured, and the mice were allowed to recover from anaesthesia. The mice were administered analgesics (buprenorphine, 10 μg/kg) and were maintained in a warm room. Seven days post-ligation, the mice were terminally anaesthetised with an overdose of pentobarbitone. Ligated and contralateral glands were dissected and weighed before analysis at the end of the experiment.

The submandibular glands were also collected and weighed post-dissection from the unoperated glands.

### 2.3. Salivary Gland Stem/Progenitor Cells Isolation and Culture

Salivary glands stem/progenitor cells (primitive cells) were isolated and cultured according to the protocols of Pringle et al. [24]. Submandibular glands were chopped and digested using a mixture of hyaluronidase (40 mg/mL), collagenase II (23 mg/mL), and CaCl_2_ (50 mM). The digestion was performed twice for 20 min in a gentle mechanical movement at 37 °C in a buffer containing 1% BSA/HBSS. Following digestion, the homogenate was washed twice with the 1% BSA/HBSS buffer and filtered. Next, the cell suspension was plated in a non-coated 12-well plate in a medium containing DMEM/F12, penicillin/streptomycin (1%), glutamax (1%), EGF (20 ng/mL), FGF (20 ng/mL), N_2_ supplement (1/100), insulin (10 μg/mL), and dexamethasone (1 μM).

All reagents were purchased from Sigma-Aldrich (Dorset, UK), except HBSS, glutamax, collagenase II and N2 supplements, which were purchased from GIBCO (Thermo Fisher Scientific Inc., Carlsbad, CA, USA).

Weights of ligated and contralateral glands were considered, and the volume of the digestion mixture was calculated for a tissue weight of 80 mg. Cells were plated according to the gland weights to maintain similar seeding densities, as ligation was performed in one gland/animal.

Unoperated (control), ligated, and contralateral glands were processed for salisphere culture. Isolated cells from unoperated glands were categorised into the following three groups for salisphere culture: the first group was for salisphere collection at four time points (*n* = 4), the second group was for rapamycin and LiCl treatments (*n* = 6), and the third group was for a collagen/matrigel culture (*n* = 4).

Ligated and contralateral glands were also processed for salisphere culture (*n* = 5).

### 2.4. Rapamycin and LiCl Treatments

Isolated cells from unoperated glands were directly treated with 22 nM of rapamycin, 10 mM of LiCl, and co-treated with rapamycin and LiCl. Salispheres were collected at different time points and homogenised with RIPA buffer (Sigma-Aldrich, Dorset, UK) for analysis.

### 2.5. Measurements of the Number and Size of Salispheres

Salispheres were imaged by performing phase-contrast microscopy (Olympus cooperation, Waltham, MA, USA). The number of salispheres was counted manually in 10 μL of medium containing salispheres from 3 wells. The area size of the salispheres was determined from 3–5 images at a magnification of 40× using Image J software version 1.46 (NIH, Maryland, MD, USA).

### 2.6. Western Blotting

Samples were processed for SDS-PAGE under reducing conditions. Protein separation was carried out using NUPAGE 4–12% Bis-Tris gel (Life Technologies, Paisley, UK) for 32 min at 200 V and 125 mA. Next, the proteins were electroblotted onto 0.45 μm nitrocellulose membranes (Anderman and Co., Kingston-Upon-Thames, UK) for one hour at 30 V and 200 mA. Proteins transferred to the nitrocellulose membranes were then processed for immunoprobing. The membrane was blocked with the Tris-buffered saline tween 20 (TBST) for one hour, followed by overnight immunoprobing with the primary antibody. Next, the membrane was washed with TBST before immunoprobing with the secondary antibody for one hour. Membranes were visualised using the ChemiDoc imaging system (Bio-Rad Laboratories Ltd., Hertfordshire, UK) with the Western C Chemiluminescent kit (Bio-Rad Laboratories Ltd., Hertfordshire, UK). All running buffers, transfer buffers, and gels were purchased from Novex (Invitrogen, Carlsbad, CA, USA).

The quantification of band intensities was performed with Image Lab software (Bio-Rad laboratories Ltd., Hertfordshire, UK), and the values of band intensities were normalised to β-actin.

### 2.7. Antibodies

For mTOR detection, anti-phospho-4e-bp1 (p-4e-bp1) and anti-phospho-S6 ribosomal protein (p-S6 rp) were used at a dilution of 1:1000 (Cell Signaling Technology, Hertfordshire, UK). β-actin was used at a dilution of 1:1500 as a housekeeping gene (Sigma-Aldrich, St. Louis, MO, USA).

The secondary antibodies applied during immunoblotting were polyclonal goat anti-rabbit immunoglobulin-HRP and polyclonal goat anti-mouse immunoglobulin-HRP, purchased from Dako Ltd. (Ely, UK).

### 2.8. Organoid Formation Assay

Salispheres were pelleted at 400 g for 5 min, resuspended with collagen and matrigel matrix (BD Bioscience, Becton, San Jose, CA, USA) at a ratio of 40:60, and plated in a 96-well plate containing 10% fetal calf serum medium. Organoids were observed and imaged every 2–3 days in culture.

### 2.9. Statistical Analysis

A statistical analysis was carried out by one-way ANOVA and Student’s unpaired *t*-test using the GraphPad Prism software Version 9.5.1 (Hampshire, UK). Data were calculated as the standard error of the mean (±S.E.M), and *p*-values less than 0.05 were considered statistically significant.

All data obtained from the untreated samples were normalised to 100% of the actual values of the area size of the salispheres, the number of salispheres, and the relative expressions for comparisons between groups. Only continuous data from growing salispheres (day 1 to day 4) were analysed according to the actual values.

## 3. Results

### 3.1. The Importance of mTOR for Salisphere Survival

**Figure 1:**
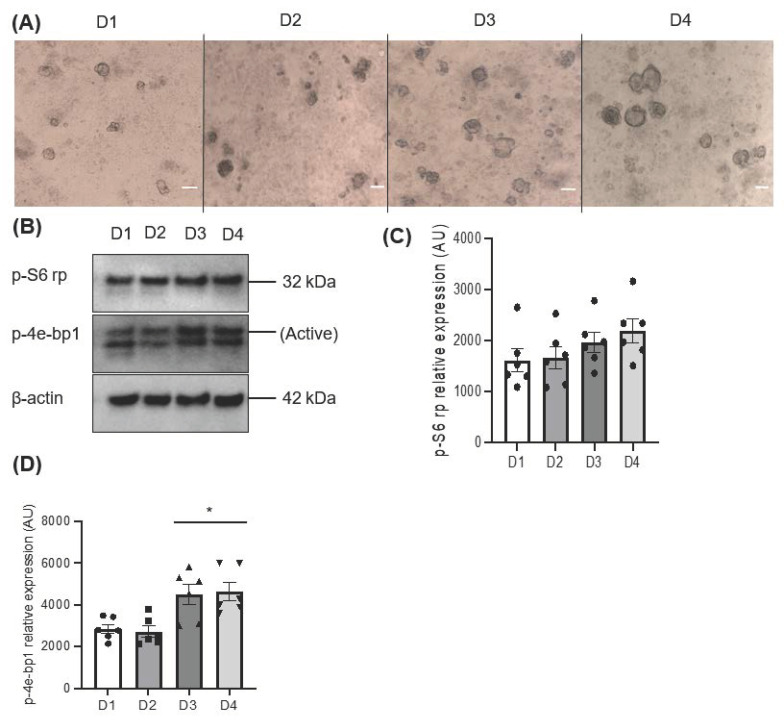
(**A**) Morphological appearance of salispheres at different time points; day 1 (D1), day 2 (D2), day 3 (D3), and day 4 (D4) of culture (scale bar = 20 µm). (**B**) Immunoblotting of mTOR substrates; p-S6 rp and p-4e-bp1 at days 1, 2, 3, and 4 of culture showed active mTOR. (**C**) Densitometric analysis reveals a gradual but insignificant increase in the phosphorylation of S6 rp (*p* = 0.2391). (**D**) A significant increase in the phosphorylation of 4e-bp1 was detected on days 3 and 4 compared to day 1 of culture (* *p* < 0.05). Data are expressed as mean ± S.E.M (*n* = 6) in arbitrary units (AUs).

A growing sphere in a serum-free condition facilitates the formation of a cluster of cells that increases in mass over time through cell–cell interactions. The morphological assessment showed a gradual increase in the area size of the salispheres (Figure 1A). The activation of mTOR was assessed by the phosphorylation of 4e-bp1 and S6 rp. The increased phosphorylation of 4e-bp1 indicates active mTOR (the upper band), while the low expression of the 4e-bp1 isoform indicates inactive mTOR (the lower band). The immunoblot showed active mTOR during the development of the salispheres (Figure 1B). Although no statistical significance was observed in the phosphorylated S6 ribosomal protein (*p* = 0.2391), a steady increase corresponding to the growth of salispheres was seen (Figure 1C). In contrast, the highest expression of p-4e-bp1 was observed on day 3 (*p* < 0.05) and day 4 (*p* < 0.05) compared to day 1 of culture (Figure 1D).

**Figure 2:**
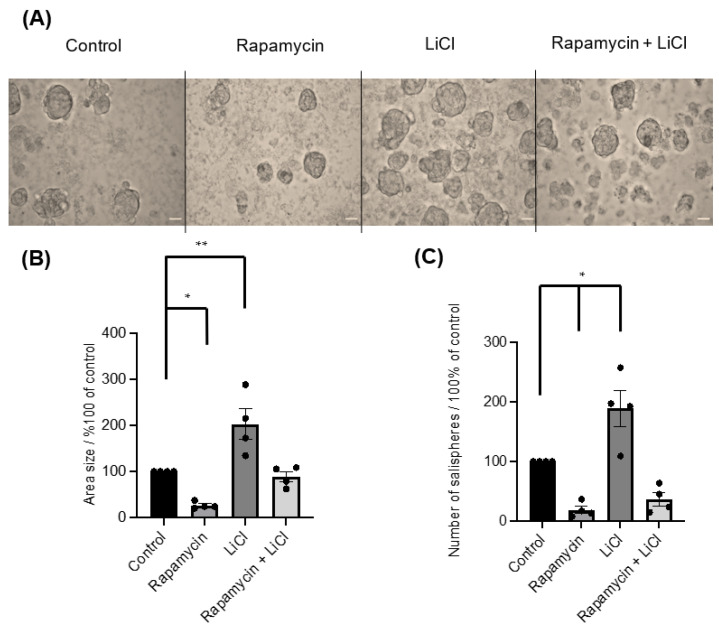
(**A**) Images of untreated salispheres (control) and salispheres treated with rapamycin, LiCl, and the combination of rapamycin and LiCl (Rapamycin + LiCl). (**B**) A significant reduction in the area size of the salispheres appeared after rapamycin (* *p* < 0.05) treatment and a significant increase in the area size appeared after LiCl treatment compared to the control (** *p* = 0.0064). A recovery in the size of the salispheres was observed in the rapamycin–LiCl cotreatment (*p* = 0.9684). (**C**) Numbers of cultured salispheres were enormously affected by rapamycin alone (* *p* < 0.05), while they improved when LiCl was combined with rapamycin (*p* = 0.0779). At the same time, a substantial increase in the number of salispheres was observed in salispheres treated with LiCl only (* *p* < 0.05) (*n* = 4). The scale bar represents 100 µm. Data are expressed as mean ± S.E.M (*n* = 4).

To investigate the importance of mTOR for salisphere formation and survival, rapamycin was applied on day 0. The inhibitor greatly affected the formation of salispheres (Figure 2A) as the area size (*p* < 0.05) (Figure 2B) and numbers (*p* < 0.05) (Figure 2C) were reduced compared to healthy growing salispheres. The use of LiCl as an indirect agonist for mTOR activation influenced the salisphere culture as it enhanced the size (*p* = 0.0064) (Figure 2B) and increased the number of salispheres (*p* < 0.05) (Figure 2C). Interestingly, a recovery in size (*p* = 0.9684) and number (*p* = 0.0779) of salispheres was observed when LiCl was combined with rapamycin.

**Figure 3:**
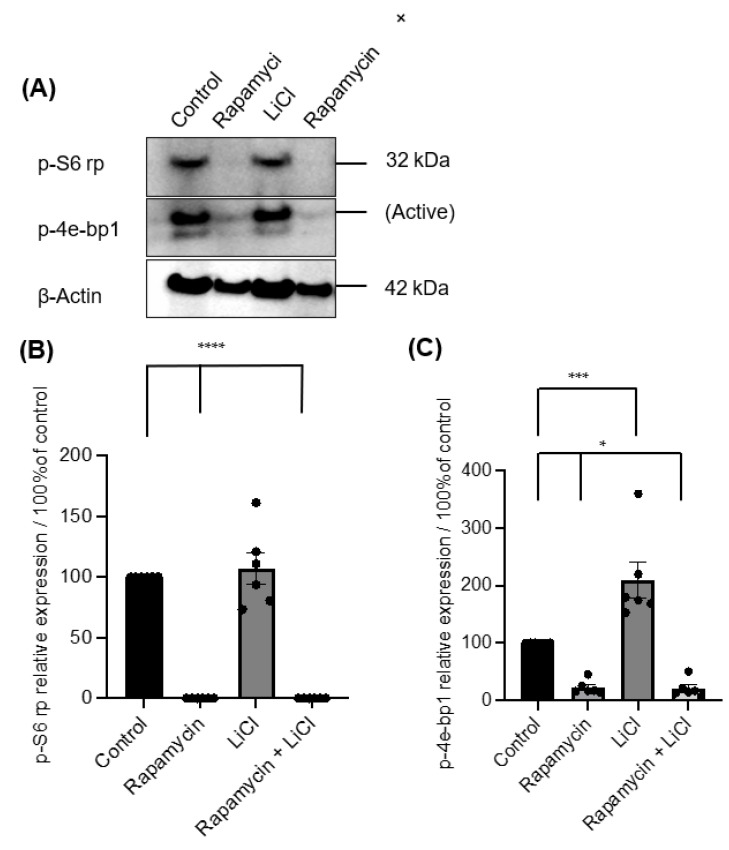
(**A**) Immunoblotting of p-S6 rp and p-4e-bp1 in untreated (control) and salispheres treated with rapamycin, LiCl, and the combination of rapamycin and LiCl (rapamycin + LiCl). (**B**). Rapamycin alone or combined with LiCl completely abolished the expression of p-S6 rp (**** *p* < 0.0001), but LiCl only did not affect the expression of p-S6 rp (*p* = 0.8898). (**C**) A considerable reduction in the phosphorylation of 4e-bp1 was observed in salispheres treated with rapamycin alone and co-treated with rapamycin and LiCl (* *p* < 0.05). In contrast, a significant increase in the phosphorylation of 4e-bp1 was detected in LiCl-treated salispheres (*** *p* = 0.0006). The bar represents the mean ± S.E.M (*n* = 6).

**Figure 4:**
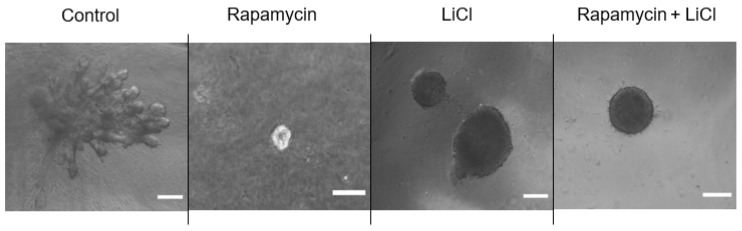
Grown salispheres in collagen:matrigel supported the branching of untreated salispheres (control) within a few days of culture. Collagen:matrigel neither assisted the recovery nor branching of salispheres in the presence of rapamycin alone. LiCl preserved the spherical structure of salispheres and assisted in the survival of salispheres and the maintenance of the spherical cluster in the presence of rapamycin. The scale bar represents 200 µm.

Immunoblotting p-S6 rp and p-4ebp1 showed variable impacts on mTOR activity after rapamycin and LiCl treatments on day four of culture (Figure 3A). Rapamycin alone or combined with LiCl completely blocked the phosphorylation of S6 rp (*p* < 0.0001) and significantly inhibited the phosphorylation of 4e-bp1 (*p* < 0.05). However, LiCl stimulated mTOR, specifically the phosphorylation of 4e-bp1 (*p* = 0.0006) compared to untreated salispheres (Figure 3B). Growing healthy salispheres in collagen:matrigel supported the development of organoids in healthy salispheres. Unlike the control, salispheres treated with rapamycin failed to survive. In addition, LiCl preserved the spherical clusters and helped the survival of salispheres in the presence of rapamycin (Figure 4).

### 3.2. The Injury Affected the Behaviour of Salispheres

**Figure 5:**
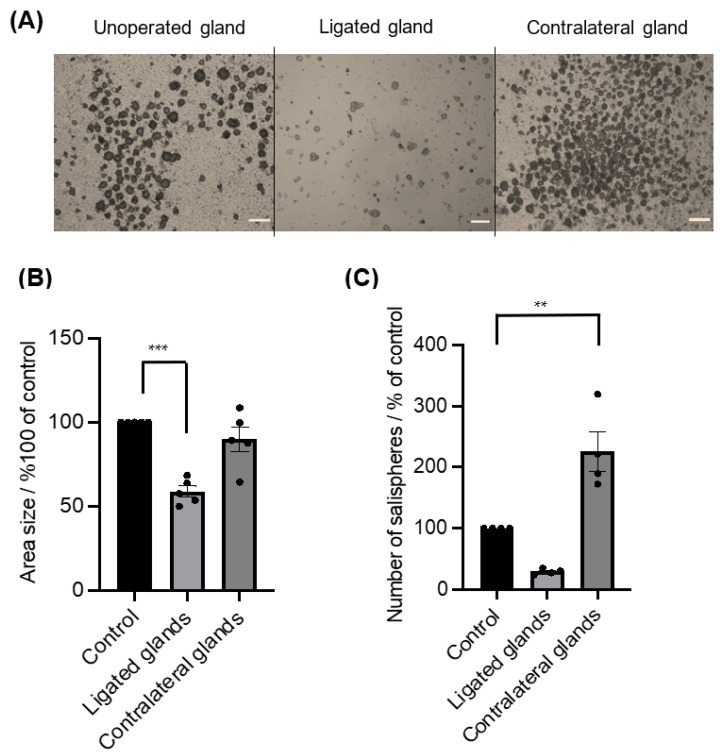
(**A**) Images of grown salispheres at day 4 of culture from unoperated glands, ligated glands, and contralateral glands (scale bar = 20 um). (**B**) The area size of the salispheres was significantly reduced in cultured salispheres from ligated glands (*** *p* < 0.0005), whereas there were no significant differences between cultured salispheres from unoperated glands and contralateral glands (*p* = 0.3251). (**C**) The number of salispheres from ligated glands was reduced, and the number of cultured salispheres from the contralateral glands was considerably higher compared to cultured salispheres from unoperated glands (** *p* < 0.005). Data are expressed as mean ± S.E.M (*n* = 5).

The ductal ligation of submandibular glands primarily affected the gland weight (40.8 ± 2.95 mg, *n* = 5) compared to the mean (63 mg, *n* = 5) of unoperated gland weights. In contrast, a substantial increase in gland weights of the contralateral gland was observed (83.2 ± 4.85 mg, *n* = 5). Ductal obstruction subsequently affected the salisphere culture (Figure 5A), as a considerable loss in size (*p* < 0.0005) (Figure 5B) and in number was observed, but with no statistical difference (*p* = 0.0637) (Figure 5C). In contrast, the contralateral glands showed a noticeable difference in the yield of salispheres compared to cultured salispheres from unoperated glands (*p* < 0.003) (Figure 5C).

**Figure 6:**
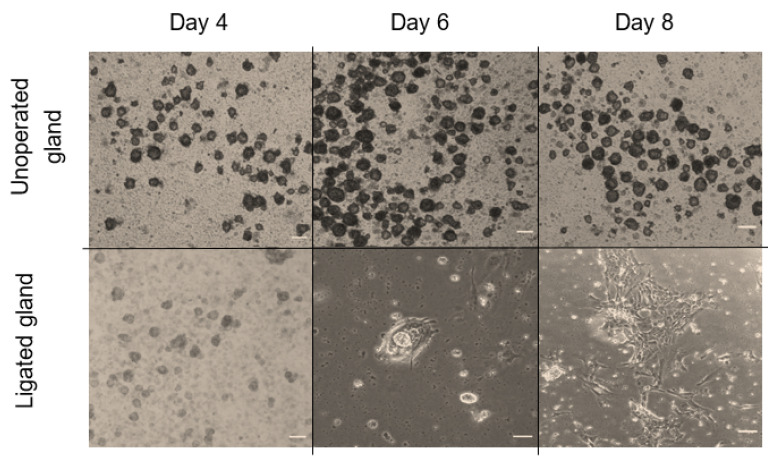
Morphological comparison between grown salispheres from unoperated and ligated glands at different time points of culture (day 4, 6, and 8) (scale bar = 10 µm). On day four of culture, salispheres appeared similar to healthy growing salispheres from unoperated glands. Grown salispheres from ligated glands behaved differently around day 6 of culture. Unlike healthy salispheres (cultured from unoperated glands), they first attach to the plastic dish (day 6), and then form fibroblastic-like structures, which increase over time of culture (day 8).

**Figure 7:**
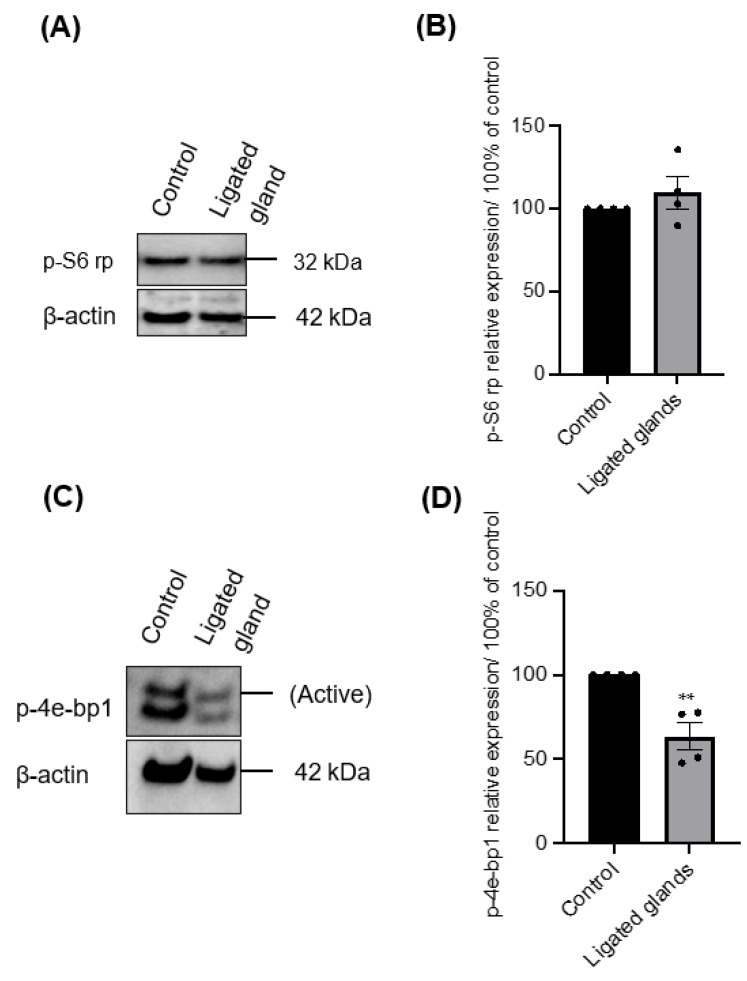
(**A**) Immunoblotting of p-S6 rp of growing salispheres from an unoperated gland (control) and from the ligated gland on day 8 of culture. (**B**) The phosphorylation of p-S6 rp in salispheres from ligated glands appeared comparable (*p* = 0.3570) to that in salispheres from unoperated glands. (**C**) Immunoblotting of p-4e-bp1 of growing salispheres from an unoperated gland (control) and from the ligated gland. (**D**) The phosphorylation of 4e-bp1 was significantly inhibited in grown salispheres from ligated glands (** *p* < 0.005). Data are expressed as mean ± S.E.M (*n* = 4).

In the first few days of culture, salispheres appeared to be similar to the control. However, they acquired a distinctive morphology by day 6 of the culture. They began to adhere to the plastic dish, form a fibroblastic-like structure, and spread over time in culture (Figure 6). Only salispheres grown from ligated glands demonstrated this feature, while salispheres grown from the contralateral glands appeared identical to the control. In addition, salispheres from ligated glands exhibited mTOR activity (Figure 7). The phosphorylated S6 rp in salispheres from ligated glands was visible and comparable to that in salispheres from unoperated glands (*p* = 0.3570) (Figure 7A,B). However, phosphorylated 4e-bp1 was considerably suppressed in cultured salispheres from ligated glands compared to that in salispheres grown from unoperated glands (*p* < 0.005) (Figure 7C,D).

## 4. Discussion

Ligation/de-ligation of the central excretory duct of the submandibular glands is a valuable model for understanding atrophy and regeneration [5,25,26]. The cross-linking between mTOR and ligation arises during atrophy. Our previous studies demonstrated that mTOR is inactive in the healthy state but becomes activated after the ductal obstruction [26,27]. It is worth noting that most research on salispheres has been performed using the irradiation model [9,14,18,28]. In this study, we used the ligation model for salisphere isolation and culture, hypothesising that the cellular response regarding mTOR might differ from the glandular response.

mTOR regulates various cellular processes, including protein synthesis and autophagy [29,30]. It has been recently reported that mTOR plays a fundamental role in the branching morphogenesis of developing salivary glands, and rapamycin can reduce the branching bud [31]. Another study revealed a positive impact of fetal bovine serum administration on the size and number of salispheres from irradiated glands [16]. Here, we first explored the role of mTOR in growing salispheres; we then assessed the effect of injury on the characteristics of salispheres, including morphology, number, and size.

The parallel relationship between mTOR activity and the size of salispheres indicated that mTOR is a key regulator of salisphere formation, as it is considered a master regulator of the cell growth [32,33]. However, it is unclear if mTOR activation was driven endogenously or by supplemented insulin in the culture medium [34].

The use of rapamycin highlighted the necessity of mTOR for salispheres’ survival. The visible phosphorylation of 4e-bp1 indicates active protein synthesis, as rapamycin did not entirely prevent salisphere formation. The rapamycin concentration used in this study was sufficient for significantly reducing the number and size of the salispheres. In contrast, another study reported a minimal effect on neurosphere formation when applying similar concentrations of rapamycin [32]. Salispheres reacted differently when rapamycin was combined with LiCl, whereby an improvement in the number and size of the salispheres was observed, yet no differences in mTOR activity were observed compared to rapamycin alone. This could suggest that the recovery of the salispheres by LiCl was driven by another signalling pathway, possibly by Wnt signals, and emphasises the necessity of mTOR activity for the full recovery of salispheres.

This suggestion is supported by the significant increase in mTOR activity and significant expansion of salispheres that occurred after LiCl treatment only. The ability of LiCl to stimulate mTOR expression implies that the increase in size and number of salispheres was probably caused to some extent by mTOR via glycogen synthase kinase-3 (GSK-3) [35]. However, Wnt possibly participated in the expansion of salispheres as LiCl is a well-established agonist for Wnt signalling [36,37].

The collagen:matrigel culture allowed healthy salispheres to branch into ductal-like structures. In contrast, the response of salispheres after rapamycin or LiCl was variable. The salispheres remaining after rapamycin treatment appeared fragile as they did not survive after placing them in the collagen:matrigel culture unless LiCl was present. This highlights the effectiveness of mTOR in terms of salispheres’ survival. Interestingly, LiCl treatment prevented the branching of salispheres and preserved the spherical cluster in the collagen:matrigel culture. Many studies have shown that the self-renewal of multiple stem/progenitor cells is mediated by Wnt signalling, including that of hematopoietic stem cells, mammary stem cells, neural stem cells, and salivary gland stem cells [1,38,39,40]. Therefore, the capability of LiCl to prevent the spherical structure from branching was presumably not driven by mTOR as it is involved in cell differentiation but possibly influenced by Wnt [41,42].

Several studies have shown that the administration of growth factors such as neurotrophic factor and keratinocyte growth factor to salivary glands assist in expanding the primitive cell pool and restoring salivary function post-radiation. [13,43,44]. Other studies have shown that stimulating or inhibiting specific signalling components can improve the yield of cultured primitive cells in vitro. For instance, the inhibition of the ROCK pathway by the Y-26732 inhibitor prevents senescence and expands the number of salispheres [17,45]. In addition, the inhibition of Wnt by WRY and IWR-1 inhibitors eliminates the growth of the salispheres [1]. The positive outcome of LiCl shown in this study suggests that LiCl could be another effective molecule for improving the yield of the salispheres.

Since mTOR appeared to be essential for salisphere growth and development, we used the ligation model to explore the effects of ductal obstruction on the characteristics of salispheres and mTOR activity. In this study, ductal ligation affected the gland weight, as documented in previous findings [27], and the abundance of salispheres corresponded to the gland weights. The morphological modification of salispheres during injury could be related to histological alterations arising from ligation, as shown in other studies [26]. Despite the reduction in salispheres after seven days of ligation, surviving primitive cells during injury could be accountable for gland recovery after releasing the ligature, as indicated in de-ligation studies [5,46]. At the same time, the hyperplasia observed in the contralateral glands suggests a compensatory action due to the significant increase in salispheres compared to salispheres grown from unoperated glands [47]. However, the relationship between hyperplasia and primitive cells is unclear.

Interestingly, the distinctive morphology of salispheres after ligation raises a question about the mechanism behind this alteration. It has been reported that cytokeratins regulate the cell shape and division of ductal cells, and radiotherapy impairs the structural cytokeratins [9,48]. Another study demonstrated positive cytokeratin 18 and cytokeratin 19 expression in isolated progenitor cells from swine-ligated glands, which possibly justifies the morphological modification in salispheres post-injury [49]. However, further investigations are required to identify the possible factors responsible for this alteration. In the context of mTOR, it appears that mTOR is essential for salisphere development, even from the ligated glands [29,32,33]. The expression of the phosphorylated 4e-bp1 during the development of salispheres, rapamycin treatment, and ligation suggest that 4e-bp1 is the dominant substrate for salisphere formation. This might be attributed to the direct relationship between 4e-bp1 and the translational machinery through the phosphorylation of the eukaryotic initiation factor 4E (eif4e) [50].

## 5. Conclusions

mTOR appeared to be a fundamental factor for protein synthesis in all growing salispheres, regardless of the source. The expression pattern of p-4e-bp1 was approximately proportional to the size and the production of salispheres, indicating the importance of mTOR for the healthy growth of salispheres.

These preliminary data also suggested that mTOR inhibition by rapamycin can negatively impact salisphere survival, while LiCl assists the expansion and growth of salispheres. Although the growth mechanism of salispheres post-injury is found to be complex, it would be interesting to detect the effect of LiCl on the recovery of isolated salispheres from ligated glands. Since ligation, radiation, and aging contribute to the reduction or elimination of salisphere formation, it would be interesting to investigate the potency of LiCl-treated salisphere transplantation in recovering injured salivary glands. Therefore, the expansion of salispheres via mTOR could lead to promising clinical outcomes in terms of the regenerative potential of salivary glands.

## 6. Limitations

We based our demonstrations on morphological assessments and protein expression analysis. The ability of isolated cultured cells to form spherical structures in vitro under the appropriate conditions and the ability of salispheres to develop organoids are the leading indicators considered in these experiments. However, determining stem cell markers such as c-kit in LiCl-treated salispheres and adhesive cells from injured glands would strongly support our findings.

## Data Availability

The data used to support the findings of this study are available from the corresponding author upon reasonable request.
